# Supplementary material for: Reversion of a RND transporter pseudogene reveals latent stress resistance potential in Brucella ovis
Source: PLoS Genet. 2025 Jul 21;21(7):e1011795. doi: 10.1371/journal.pgen.1011795 (PMC12306736; doi:10.1371/journal.pgen.1011795)
Supplement: S1 Table — These compounds passed the initial screening criteria as selectively inhibitory to B. ovis during THP-1 macrophage infection with limited axenic activity. (PDF) [file pgen.1011795.s001.pdf]

Sample	Compound name	Therapeutic class	Major pathway or target
MSU-51	Trichlorfon	Pesticide	Acetylcholinesterase inhibitor
MSU-339	Guanfacine hydrochloride	Hypertension	Adrenergic receptor agonist
MSU-270	Fendiline hydrochloride	Antiarrhythmic	Calcium
MSU-368	Bepridil hydrochloride	Angina	Calcium
MSU-383	Nicardipine hydrochloride	Hypertension	Calcium
MSU-1264	Lomerizine hydrochloride	Migraine	Calcium
MSU-1188	Cilnidipine	Hypertension	Calcium
MSU-1180	Bifonazole	Antimicrobial	Cell membrane (fungi)
MSU-1011	Flucloxacillin sodium	Antimicrobial	Cell wall
MSU-470	Ceforanide	Antimicrobial	Cell wall
MSU-700	Cefmetazole sodium salt	Antimicrobial	Cell wall
MSU-1212	Ezetimibe	Cholesterol absorption	Cholesterol
MSU-370	Benzbromarone	Gout	Cytochrome P450
MSU-605	Carbadox	Antimicrobial	DNA synthesis
MSU-350	Clozapine	Antipsychotic	Dopamine
MSU-360	Droperidol	Nausea	Dopamine
MSU-374	Methylergometrine maleate	Uterine atony	Dopamine
MSU-980	Piribedil hydrochloride	Depression	Dopamine
MSU-1163	Aripiprazole	Antipsychotic	Dopamine, serotonin
MSU-626	Racecadotril	Diarrhea	Enkephalinase
MSU-976	Tracazolate hydrochloride	Sedative	GABA
MSU-457	Meclozine dihydrochloride	Nausea	Histamine
MSU-589	Azelastine HCl	Allergic conjunctivitis	Histamine
MSU-888	Promethazine hydrochloride	Allergy	Histamine
MSU-1260	Ritonavir	HIV	HIV protease
MSU-973	Pirlindole mesylate	Depression	Monoamine oxidase
MSU-173	Tranylcypromine hydrochloride	Depression	Monoamine oxidase
MSU-144	Loperamide hydrochloride	Diarrhea	Mu-opioid receptors
MSU-581	Reboxetine mesylate	Depression	Noradrenaline
MSU-1211	Ipriflavone	Osteoporosis	Osteoclast
MSU-376	Clofazimine	Leprosy	Peroxisome

MSU-587	Cilostazol	Vasodilator	Phosphodiesterase
MSU-977	Zardaverine	Cancer	Phosphodiesterase
MSU-142	Dipyridamole	Anticoagulant	Phosphodiesterase
MSU-1031	Halofantrine hydrochloride	Malaria	Porphyrin
MSU-533	Phenacetin	Analgesia	Prostaglandin
MSU-660	Avermectin B1a	Antimicrobial	Protein synthesis
MSU-476	Primaquine diphosphate	Malaria	Reactive oxygen species
MSU-1105	Verteporfin	Macular degeneration	Reactive oxygen species
MSU-590	Etretinate	Psoriasis	Retinoic acid
MSU-531	Pirenperone	Anxiety	Serotonin
MSU-979	Ozagrel hydrochloride	Thrombosis	Thromboxane
MSU-853	Liothyronine	Hypothyroidism	Thyroid
MSU-494	Propylthiouracil	Hyperthyroidism	Thyroid peroxidase
MSU-381	Lidoflazine	Experimental	Unknown
MSU-421	Suloctidil	Experimental	Unknown
MSU-550	Parthenolide	Dermatitis	Unknown
MSU-1013	Deptropine citrate	Experimental	Unknown
MSU-1054	Levopropoxyphene napsylate	Cough	Unknown
