# Supplementary figures and images for: Reversion of a RND transporter pseudogene reveals latent stress resistance potential in Brucella ovis
Source: PLoS Genet. 2025 Jul 21;21(7):e1011795. doi: 10.1371/journal.pgen.1011795 (PMC12306736; doi:10.1371/journal.pgen.1011795)

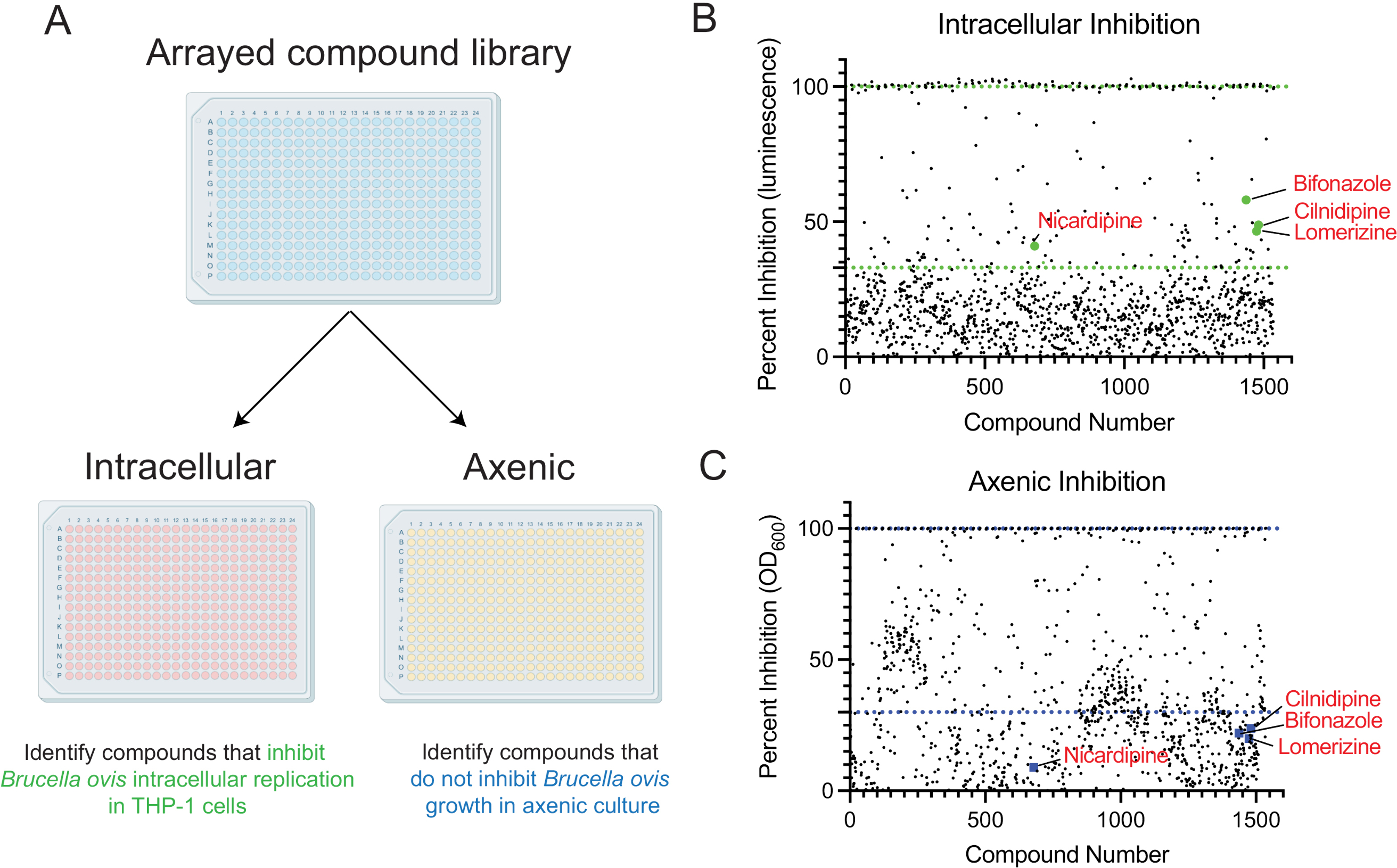

Supplement: S1 Fig — (A) Diagram of the drug screening pipeline for the identification of small molecules that selectively inhibit B. ovis intracellular growth with minimal axenic activity. (B) Intracellular inhibition of all tested small molecules, shown as percentage of luminescence emitted by B. ovis cells harboring the lux operon. Highlighted in green are drug candidates that inhibited B. ovis intracellular growth in THP-1 macrophages. The screening of the Prestwick Chemical library had a Z’ factor of 0.417 for inhibition of intracellular growth. Dotted line represents hit determination of 35% intracellular inhibition. (C) Effect of small molecules on B. ovis growth inhibition in axenic culture. The screening of the Prestwick Chemical library had a Z’ factor of 0.473 for axenic growth inhibition. Dotted line represents 30% axenic inhibition. The compounds highlighted in green in B and blue in C are hits that inhibited B. ovis intracellular growth but had minimal axenic activity based on our screening criteria. (TIF) [file pgen.1011795.s003.tif]

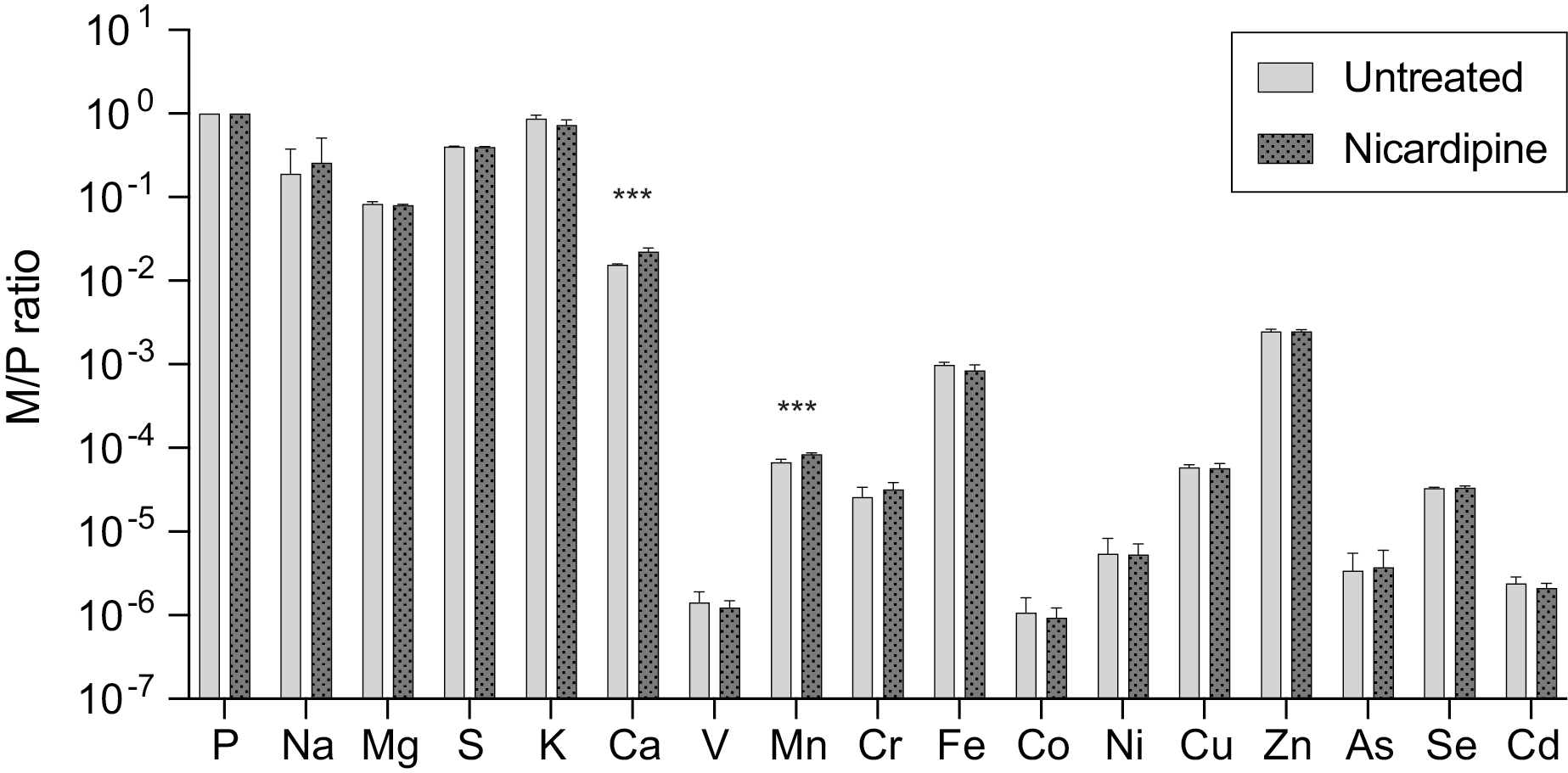

Supplement: S2 Fig — Element content was determined by triple quadrupole inductively coupled plasma mass spectrometry (ICP-QQQ). Levels of each element were normalized to total phosphorus levels (M/P). Bars represent the mean ± standard deviation of 7 biological replicates measured over 2 independent experiments. The M/P ratios for each metal were compared using multiple unpaired t-tests and the Bonferroni-Dunn method to adjust for multiple comparisons (***, adjusted P < 0.001). (TIF) [file pgen.1011795.s004.tif]

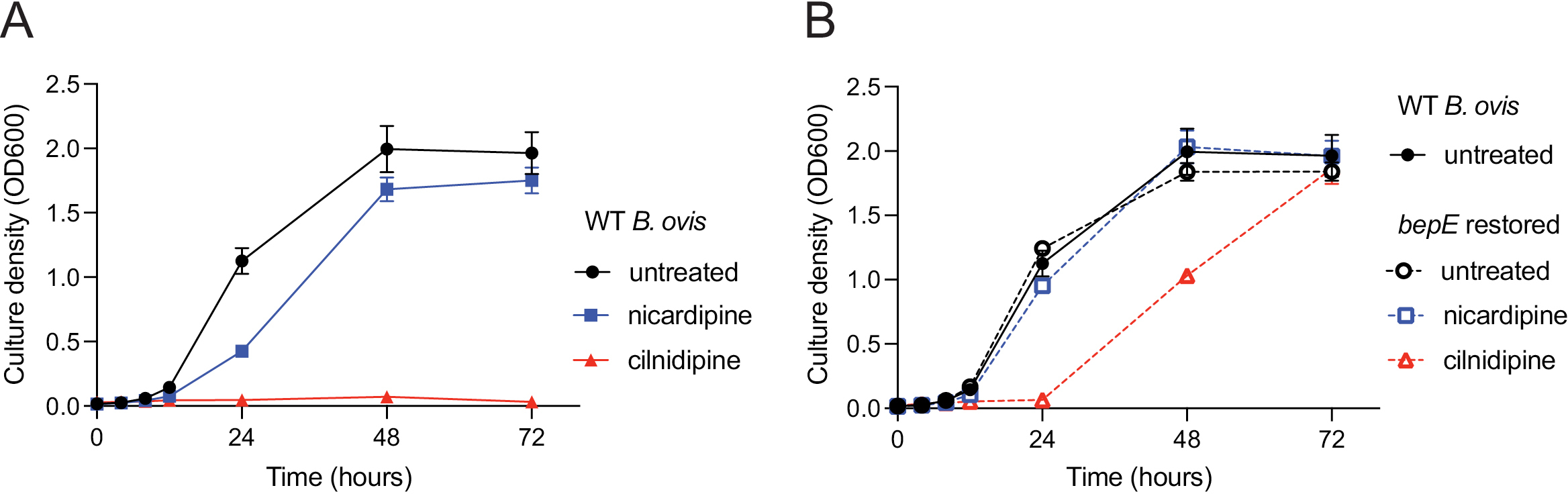

Supplement: S3 Fig — (A) Growth of wild-type (WT) B. ovis cultures, untreated (black) 25 µM nicardipine (blue) or 25 µM cilnidipine (red), was monitored by optical density at 600 nm. (B) Growth of the bepE restored strain in the same treatments as in (A). The WT untreated culture is presented in both panels for reference. (TIF) [file pgen.1011795.s005.tif]

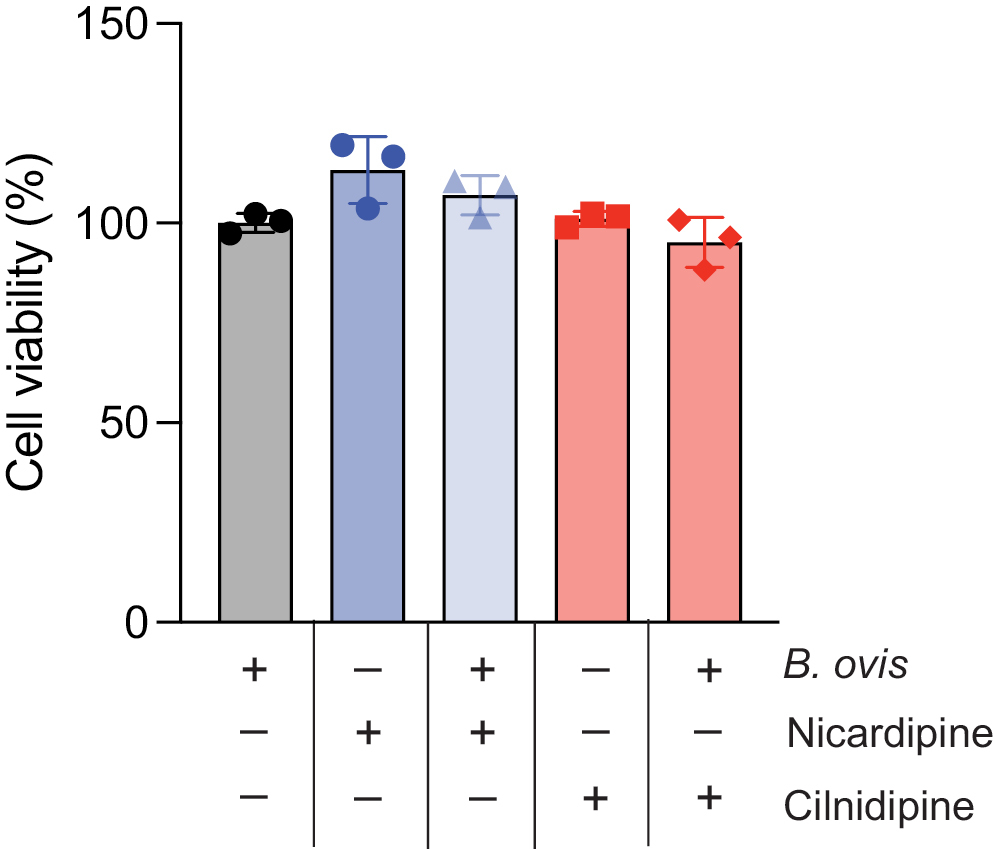

Supplement: S4 Fig — Viability of THP-1 cells, assessed at 48 h post infection or following treatment with 25 µM nicardipine or 25 µM cilnidipine with the XTT-cell proliferation assay. Viability was normalized to wells containing untreated THP-1 cells infected with B. ovis and to wells only containing cell culture medium (blank). Values are means ± SD from three independent trials. (TIF) [file pgen.1011795.s006.tif]

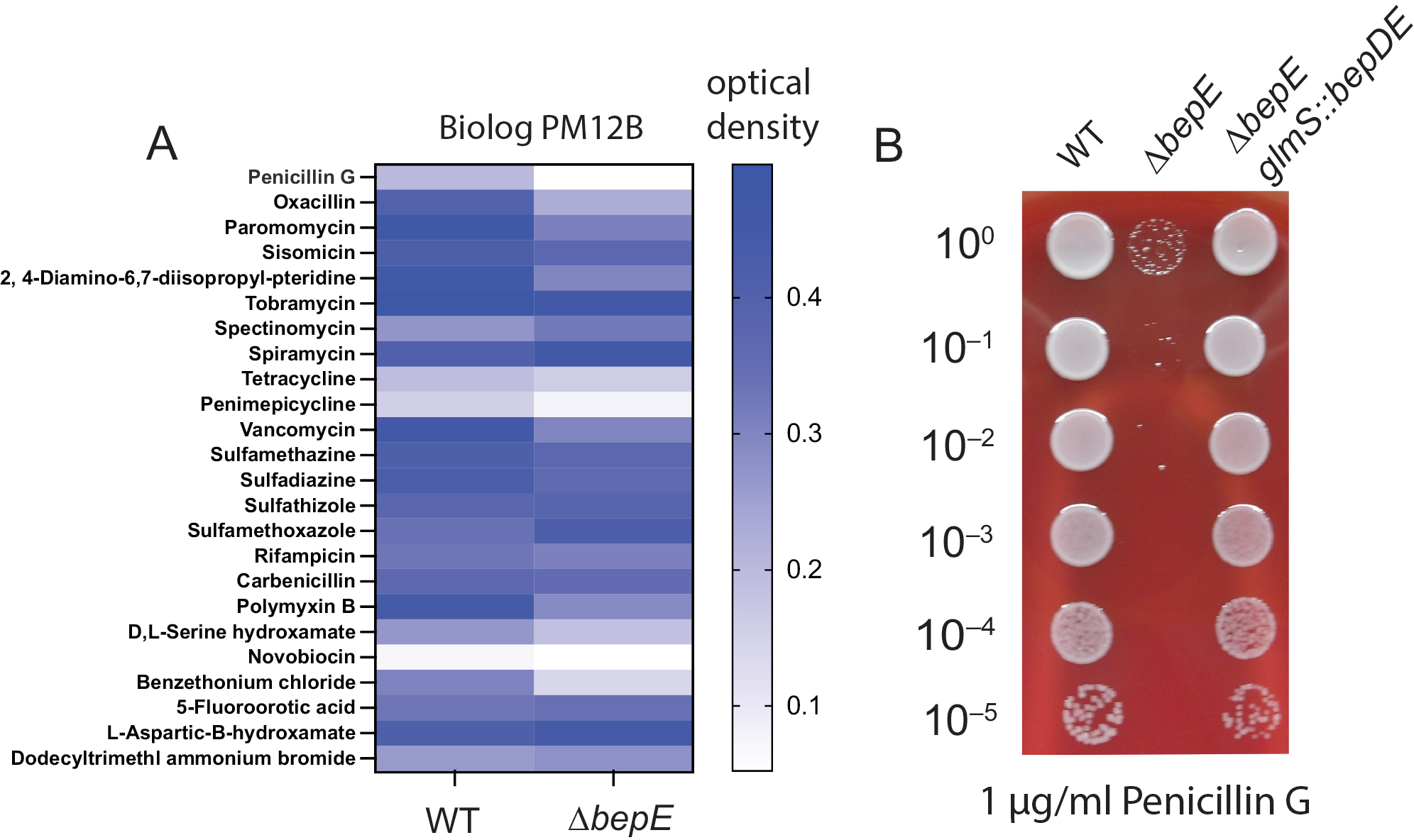

Supplement: S5 Fig — (A) Heat-map of WT and ∆bepE growth (optical density at 600 nm) in Biolog phenotype microarray plate PM12B. (B) Growth of serially-diluted B. abortus strains spotted onto TSA blood plates with 1 µg/ml penicillin G (w/v). (TIF) [file pgen.1011795.s007.tif]

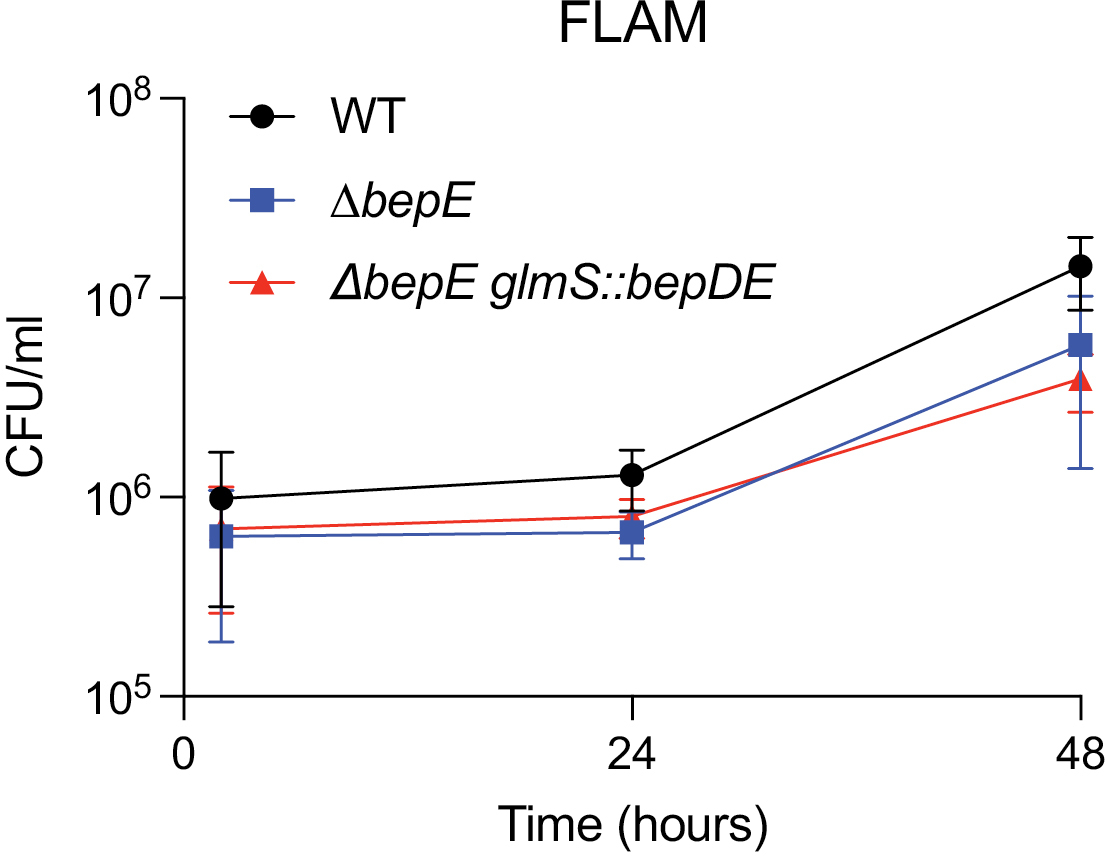

Supplement: S6 Fig — bepE does not contribute to B. abortus survival in fetal liver alveolar macrophages (FLAM). B. abortus (WT, ∆bepE, and the complementation strain ∆bepE glmS::bepDE) recovered from FLAM cells after infection. Values are means ± SD CFU recovered from three independent trials. (TIF) [file pgen.1011795.s008.tif]

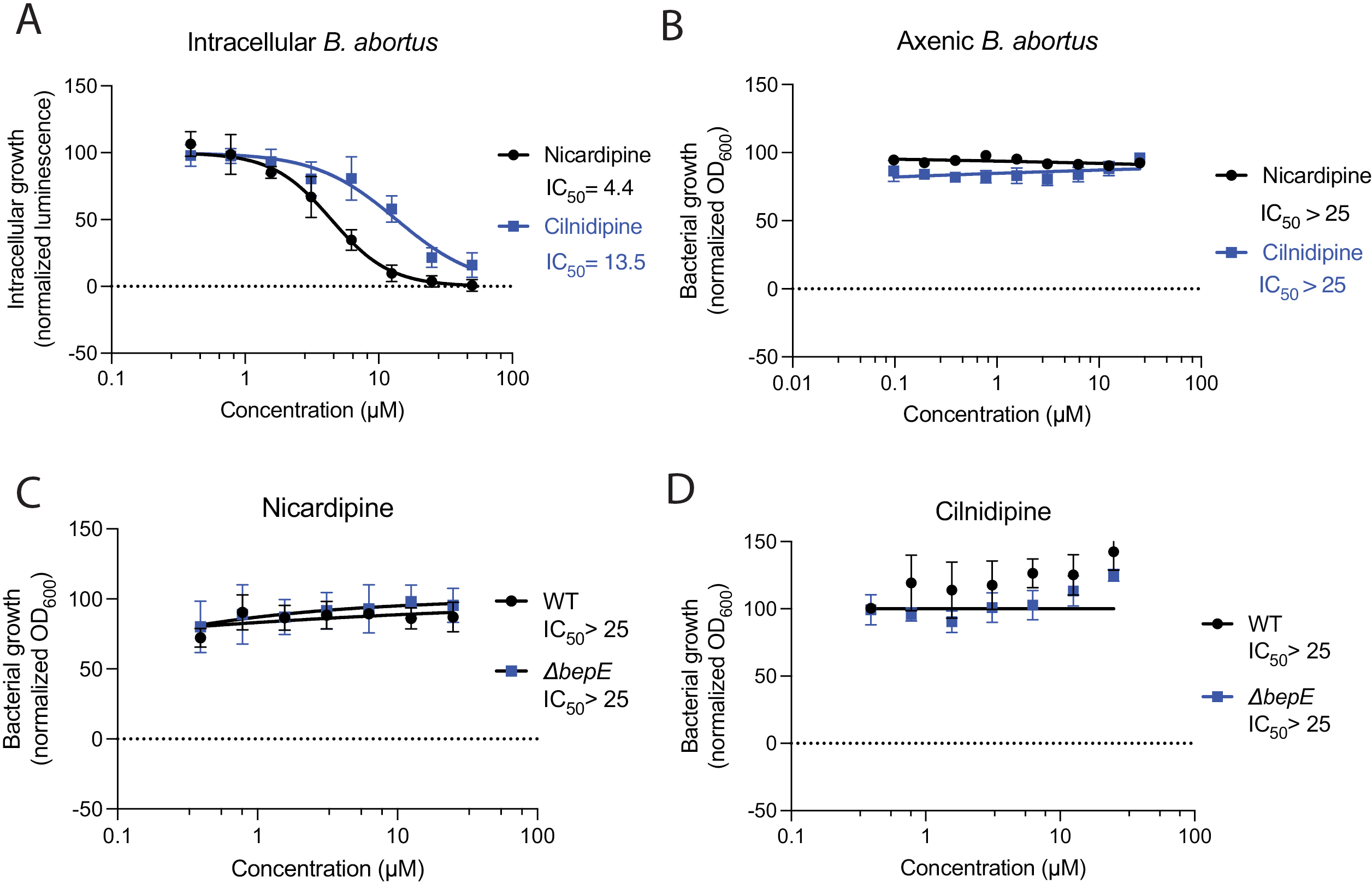

Supplement: S7 Fig — (A) Intracellular inhibitory activities of nicardipine or cilnidipine during infection of THP-1 macrophages by lux expressing B. abortus. Luminescence was measured after 48 h and normalized to untreated infected controls. (B) Axenic inhibitory activity of nicardipine and cilnidipine during B. abortus WT growth in liquid medium. Optical density at 600 nm was measured at 48 h and normalized to untreated cultures. (C, D) Disruption of bepE does not affect axenic sensitivity of B. abortus to nicardipine or cilnidipine. Growth was measured and analyzed as in panel B. (TIF) [file pgen.1011795.s009.tif]
